# Volumetric‐based image guidance is superior to marker‐based alignments for stereotactic body radiotherapy of prostate cancer

**DOI:** 10.1002/acm2.12280

**Published:** 2018-02-15

**Authors:** Wen Li, Lan Lu, Kevin L Stephans, Naveen Sharma, Andrew Vassil, Zhilei Liu Shen, Abigail Stockham, Toufik Djemil, Rahul D Tendulkar, Ping Xia

**Affiliations:** ^1^ Department of Radiation Oncology Cleveland Clinic Foundation Cleveland OH USA

**Keywords:** endorectal balloon, kV‐CBCT, prostate, SBRT

## Abstract

**Purposes:**

The aim of this study was to evaluate a dual marker‐based and soft‐tissue based image guidance for inter‐fractional corrections in stereotactic body radiotherapy (SBRT) of prostate cancer.

**Methods/Materials:**

We reviewed 18 patients treated with SBRT for prostate cancer. An endorectal balloon was inserted at simulation and each treatment. Planning margins were 3 mm/0 mm posteriorly. Prior to each treatment, a dual image guidance protocol was applied to align three makers using stereoscopic x ray images and then to the soft tissue using kilo‐voltage cone beam CT (kV‐CBCT). After treatment, prostate (CTV), rectal wall, and bladder were delineated on each kV‐CBCT, and delivered dose was recalculated. Dosimetric endpoints were analyzed, including V_36.25 Gy_ for prostate, and D_0.03 cc_ for bladder and rectal wall.

**Results:**

Following initial marker alignment, additional translational shifts were applied to 22 of 84 fractions after kV‐CBCT. Among the 22 fractions, ten fractions exceeded 3 mm shifts in any direction, including one in the left‐right direction, four in the superior‐inferior direction, and five in the anterior‐posterior direction. With and without the additional kV‐CBCT shifts, the average V_36.25 Gy_ of the prostate for the 22 fractions was 97.6 ± 2.6% with the kV x ray image alone, and was 98.1 ± 2.4% after applying the additional kV‐CBCT shifts. The improvement was borderline statistical significance using Wilcoxon signed‐rank test (*P* = 0.007). D_0.03 cc_ was 45.8 ± 6.3 Gy vs. 45.1 ± 4.9 Gy for the rectal wall; and 49.5 ± 8.6 Gy vs. 49.3 ± 7.9 Gy for the bladder before and after applying kV‐CBCT shifts.

**Conclusions:**

Marker‐based alignment alone is not sufficient. Additional adjustments are needed for some patients based kV‐CBCT.

## 1 INTRODUCTION

Stereotactic body radiation therapy (SBRT) is a promising treatment regimen for localized prostate cancer because of the low *α*/*β* ratio of the prostate adenocarcinoma.^1^ Several recent SBRT studies showed local control rates and toxicity profiles were comparable to the conventional dose regimen of 2 Gy per fraction (2 Gy/Fx).^2^, ^3^ It has been reported that with a prescription dose of 7.25 Gy/Fx or higher in SBRT treatment planning, margins are usually 5 mm or smaller to spare the surrounding organs at risk such as rectum, urethra, and bladder.^1^ The high‐dose conformity of SBRT plans imposes a stringent requirement to the management of inter‐ and intra‐fractional variations.

Intra‐fractional motion can be managed with use of an endorectal balloon.^4^, ^5^, ^6^, ^7^, ^8^ However, it may introduce a large prostate inter‐fraction motion, rotation, and deformation. Jones et al. reported that 69% of fractions required insertion adjustments of the endorectal balloon to reduce prostate rotation and deformation.^6^ They recommended to acquire two Cone‐Beam Computed Tomography (CBCTs) for each patient: one after insertion and the other after adjustment. With daily Image‐guided Radiation Therapy (IGRT) to correct translational setup error and inter‐fractional motion of the prostate, uncorrected prostate rotation became a predominant impact on the dose delivered to the prostate. With 2‐, 3‐, and 5‐mm Planning Target Volume (PTV) margins, Amro et al. showed that only 39%, 65%, and 84% of 26 patients had adequate dose coverage to the prostate without rotation correction, respectively.^9^

With a tight planning margin, we implemented a two‐step (dual) IGRT protocol for patients receiving prostate SBRT treatment with endorectal balloon. The dual IGRT protocol involved with a marker‐based kV x ray guidance and six dimensional (6D) corrections using a robotic couch, and a soft‐tissue based kV‐CBCT with 3D translational corrections. Using a dosimetric analysis, the aim of this study was to evaluate whether the dual marker‐based and soft‐tissue based IGRT protocol is sufficient to achieve adequate Clinical Target Volume (CTV) coverage.

## 2 MATERIALS AND METHODS

Eighteen patients treated with five‐fraction SBRT for prostate cancer were included in this study. Patients were implanted with three gold fiducial markers at apex, left and right base of their prostate glands. A total of 84 daily dose fractions were included in the dosimetric analysis (six fractions were not included due to incomplete imaging of the anatomical volume). In 22 of 84 fractions, additional translational corrections guided by kV‐CBCT were applied after the kV x ray guided 6D shifts. These 22 fractions were separately analyzed to evaluate dosimetric differences between with and without the additional kV‐CBCT shifts after the 6D kV x ray guided shifts.

To minimize the position variations of the internal organs, the patients were instructed to maintain a full bladder and empty rectum before the simulation and treatments. Immediately before the acquisition of the simulation CT and the daily imaging, an endorectal balloon was inserted and filled with 80–100 cc air to immobilize the prostate. Patients were setup in the supine position in a vac‐lok bag (Civco Medical Solution, Coralville, IA, USA). After an initial laser alignment to the patients' skin tattoos, daily two‐step IGRT protocol was performed. First, a 6D shift was applied using a robotic couch (ExacTrac, Brainlab, Feldkirchen, Germany) according to marker registration on the daily kV x ray images from ExacTrac. A kV‐CBCT then verified soft‐tissue prostate localization. The additional 3D couch translational shift was applied if deemed necessary, for instance, if the shift at any of direction was larger than 2 mm. The additional rotational shifts from the kV‐CBCT were ignored since the CBCT was not interfaced with the 6D robotic couch.

**Figure 1:**
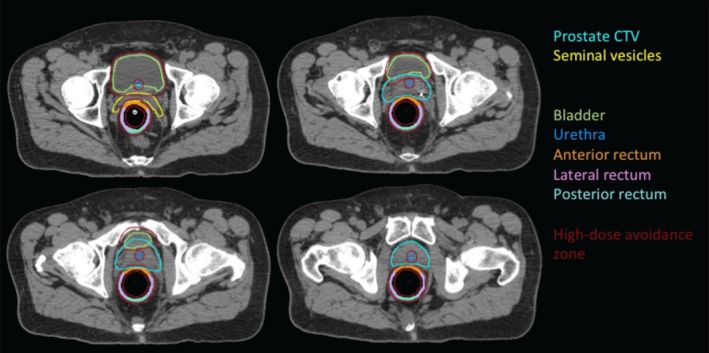
An example of prostate SBRT contours, including high‐dose avoidance zone, prostate CTV, seminal vesicles, bladder, urethra, and segmented rectum.

All patients were planned according to our in‐house protocol approved by our local institutional review board (IRB). Briefly, the prostate (CTV) and seminal vesicle (SV) were delineated as the treatment target, and rectum, urethra and bladder were delineated as organs at risks (OAR) according to our protocol. We defined high‐dose avoidance zone (HDAZ) with 3 mm expansion of those OAR (Fig. 1). PTVs included low‐dose PTV (LD‐PTV) and high‐dose PTV (HD‐PTV). The LD‐PTV was a 3 mm uniform expansion of the prostate CTV (0 mm posteriorly) and seminal vesicle in all directions. The HD‐PTV consisted of LD‐PTV subtracting HDAZ. The prescription doses were 50 Gy to HD‐PTV and 36.25 Gy to LD‐PTV in 5 fractions. All treatment plans were created in Pinnacle 9.0 (Philips, Fitchburg, WI, USA) using volumetric modulated arc therapy (VMAT) (2 or 4 full arcs). The planning dose constraints included ≥95% of the LD‐PTV volume to receive ≥36.25 Gy (V_36.25 Gy_ ≥95%) and ≥99% of the prostate CTV to receive ≥36.25 Gy (V_36.25 Gy_ ≥99%). For the HD‐PTV, a mean dose of 50 Gy or higher was desired but had a lower priority than OAR constraints. Dose constraints for the anterior rectal wall included a maximum dose (D_0.03 cc_) less than 50 Gy (D_0.03 cc_ ≤50 Gy) and maximum dose to 1 cc of the anterior rectal wall (D_1 cc_) less than 45 Gy (D_1 cc_ ≤45 Gy). The maximum dose to 0.03 cc of the bladder was limited to 105% of prescription dose (D_0.03 cc_ ≤52.5 Gy). The maximum dose to urethra was restricted to 50 Gy (D_0.03 cc_ ≤50 Gy).

**Figure 2:**
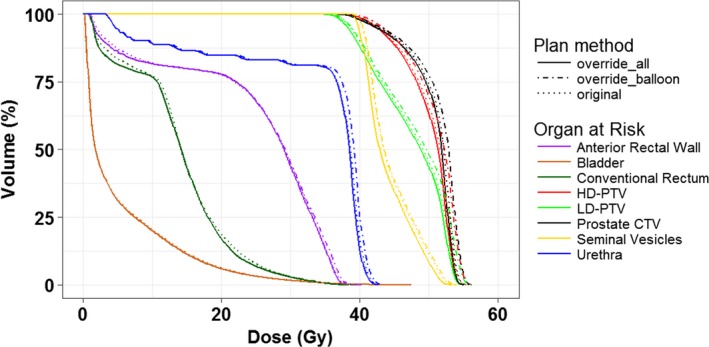
For a selected patient, compassion of three dose volume histograms: (a) override all tissue with an electron density of 1 g/cm^3^ (override all); (b) override the rectal balloon with an electron density of 0 g/cm^3^ while other tissue with electron density of 1 g/cm^3^ (override balloon); (c) the original plan with no overrides.

On each daily verification kV‐CBCT, the prostate (CTV), rectal balloon, and bladder were retrospectively delineated by the attending physicians. To remove the effect from inaccurate electron density information in kV‐CBCT, an external contour was created for both planning CTs and daily kV‐CBCTs in which a uniform electron density of 1 g/cm^3^ was assigned. To understand dosimetric differences between the heterogeneity and homogeneity dose calculations on a selected patient (Fig. 2), we compared dose volume histograms (DVH) (a) override all tissue with a uniform electron density of 1 g/cm^3^ (override all); (b) override the rectal balloon with electron density of 0 g/cm^3^ while other tissue with electron density of 1 g/cm^3^ (override balloon); (c) with heterogeneity dose calculation. The differences were less than 2%. To provide a reference for daily dose evaluation, the planning dose was then recalculated with the uniform electron density. On daily kV‐CBCT, dose was calculated applying the original treatment plan with the treatment iso‐center. For the 22 fractions that had additional kV‐CBCT‐guided shifts, to compare dosimetric outcomes with and without additional kV‐CBCT‐guided shift, the dose was also calculated with the beam iso‐centers positioned prior to the kV‐CBCT shifts.

For the evaluation of daily target coverage, the prostate (CTV) V_36.25 Gy_ was used since the planning goal on the HD‐PTV coverage was not a priority. The D_0.03 cc_ of the rectal wall was used to evaluate the daily dose toxicity. The rectal wall was created by placing a 2‐mm thick ring just outside the rectal balloon surface on both daily kV‐CBCTs and planning CTs. The bladder D_0.03 cc_ was used to evaluate daily bladder dose.

Descriptive statistics were used for data analysis. The results were expressed as mean ± standard deviation. Wilcoxon signed rank test was used to compare the daily dosimetric endpoints between using the kV x ray guided shifts alone and with the additional kV‐CBCT guided shifts. Statistical significance was assigned at *P* < 0.05.

## 3 RESULTS

For the eighteen patients, all original treatment plans with homogeneity dose recalculation met planning constraints. The average V_36.25 Gy_ of the prostate CTV was 100.0 ± 0.0% (range: 99.99%–100%), the average bladder D_0.03 cc_ was 45.8 ± 3.5 Gy (range: 38.7–51.8 Gy), and the average rectal wall D_0.03 cc_ was 42.1 ± 3.1 Gy (range: 39.2–49.0 Gy).

**Figure 3:**
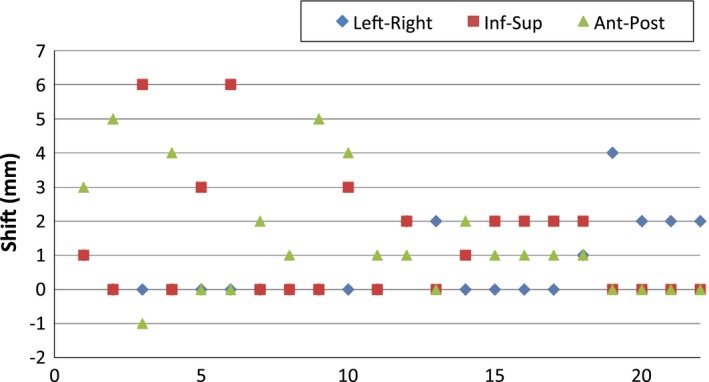
A plot of detailed shifts in the left‐right, inferior‐superior, and anterior‐posterior directions for 22 fractions.

Among 84 fractions, additional translational shifts were made at the discretion of the treatment team in 26% of the fractions (22 of 84) for 50% of the patients (nine of 18). The specific directional shifts from these 22 fractions are plotted in Fig. 3. Among the 22 fractions, 10 fractions exceeded 3 mm shifts in any direction, including one in the left‐right direction, four in the superior‐inferior direction, and five in the anterior‐posterior direction.

**Figure 4:**
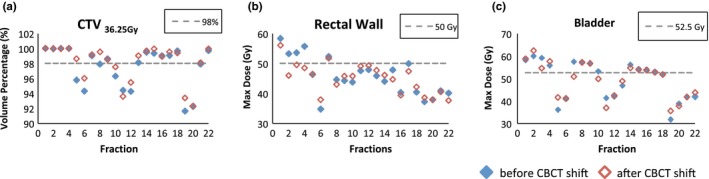
Dosimetic endpoints of V_36.25 Gy_ for the prostate CTV (a), rectal wall D_0.03 cc_ (b), and bladder D_0.03 cc_ (c) before and after CBCT correction for the 22 fractions.

For each of these 22 fractions, two separate plans were generated with and without the additional kV‐CBCT‐guided shifts. With only kV x ray guided 6D corrections, the average V_36.25 Gy_ of the CTV was 97.6 ± 2.6%. After applying the additional kV‐CBCT guided shifts, the average V_36.25 Gy_ increased to 98.1 ± 2.4%. The improvement was borderline statistical significance using Wilcoxon signed rank test (*P* = 0.007). Figure 4(a) shows the details of CTV coverage changes for 22 individual fractions before and after kV‐CBCT correction. For the OARs, a trend of decease in the maximum doses (D_0.03 cc_) was observed but not statistically significant for both the rectal wall and bladder. For instance, the average D_0.03 cc_ of the rectal wall was 45.8 ± 6.3 Gy and 45.1 ± 4.9 Gy, respectively, before and after applying the additional shifts (*P* = 0.41). The average D_0.03 cc_ of bladder were 49.5 ± 8.6 Gy and 49.3 ± 7.9 Gy, respectively, before and after the additional shifts (*P* = 0.95).

## 4 DISCUSSION

In this study, we evaluated a dual marker‐based and soft‐tissue based IGRT protocol in compensating inter‐fraction error in prostate SBRT. Our results demonstrate that, after marker‐based kV x ray guidance and 6D corrections, additional translational adjustments based on soft‐tissue alignment on kV‐CBCT are needed in 26% of the fractions (22 of 84), indicating that marker‐based alignment alone is not sufficient, CBCT‐based soft‐tissue alignment with implanted marker guidance is superior to the marker alignment alone.

Differences between the soft‐tissue alignment using kV‐CBCT and the fiducial marker alignment using 2D kV x ray have been reported in several studies.^10^, ^11^, ^12^ Using independent soft‐tissue alignment and marker alignment for 36 patients in 286 images, Barney et al. reported 28% of fractions had a difference greater than 5.0 mm at one or more dimensions.^10^ Using kV‐CBCT for the soft‐tissue alignment and 2D kV projection images from the kV‐CBCT for the marker alignment for fifteen patients in 256 images, Moseley et al. reported agreement within 3 mm between the two alignment methods in 90.8%, 63.7%, and 64.1% fractions at the lateral, vertical, and longitudinal directions.^12^ These results are in general agreement with our results, although we used two‐step alignments instead of two independent alignments.

Several studies^7^, ^8^ indicated that utilizing an endorectal balloon can effectively immobilize prostate and restrict rectal filling. The use of endorectal balloon, however, may introduce a large inter‐fraction prostate motion and deformation.^4^ Neither translational nor rotational correction can resolve issues of organ deformation and organ shape changes. Therefore, online adaptive replanning strategies^13^, ^14^, ^15^, ^16^ may provide an optimal solution while maintaining tight planning margins. Without online adaptive planning, alternative strategy is to minimize inter‐fractional prostate deformation and rotation. Instead of inserting the rectal balloon, one can use the transperineal ultrasound to monitor intra‐prostate motion. Instead of using implanted markers as the surrogate of the prostate, one can directly align to the prostate soft tissue.

One limitation of this study is no consideration of the intra‐fractional variation, although other studies have shown the effectiveness of prostate immobilization with the use of rectal balloon.^4^, ^7^, ^8^ A recent prostate SBRT study also showed that the compensation of intra‐fractional motion on a beam‐by‐beam basis has very little impact on the final dose parameters.^16^ Therefore, we believe that the prostate motion between the two IGRT acquisition time delta is negligible.

**Figure 5:**
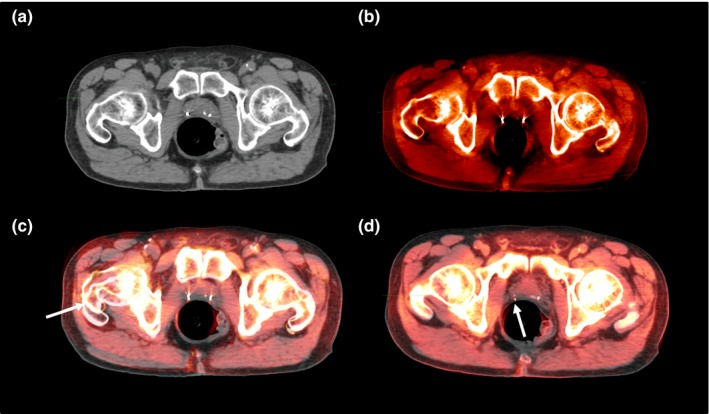
For a selected patient, (a) an axial planning image with two implanted markers; (b) the corresponding axial kV‐CBCT image as (a); (c) marker‐based alignment of images (a) and (b); (d) soft‐tissue based alignment of image (a) and (b). (Arrows indicate the misalignments).

It has been reported that alignment to markers often results in a larger rotation than with soft‐tissue alignment.^17^, ^18^ The detected average rotation were 1.87^o^ ± 2.60^o^ (range: −6.10^o^ to 8.14^o^), 1.31^o^ ± 1.55^o^ (range: −2.26^o^ to 5.70^o^) 0.51^o^ ± 0.63^o^ (range: −1.81^o^ to 1.98^o^) in yaw, roll, and pitch, respectively. Figure 5 shows an example of marker‐based alignment [Fig. 5(c)] compared to soft‐tissue alignment with implanted marker guidance [Fig. 5(d)]. Figures 5(a) and 5(b) are planning CT and kV‐CBCT, respectively, to display the marker locations prior to image fusion. In Fig. 5(c), a greater misalignment of soft‐tissue and bony structure rotation were observed although the implanted markers were perfectly aligned. From Fig. 5(d), under implanted marker guidance, the alignment of the prostate was improved and the large body rotation was eliminated. Meanwhile, the markers in Fig. 5(d) were a few mm misaligned due to potential prostate deformation. It is worthy of noticing that with a newly integrated 6D couch with kV‐CBCT, we no longer use this dual process but use implanted marker as guidance to align prostate soft tissue in kV‐CBCTs and in the planning CTs. In this report, because of limited 6D corrections in current commercially available treatment couch, prostate rotations are not fully compensated, which could be one of the reasons for sub‐optimal dose coverage for the treatment targets. Once the confidence in soft‐tissue alignment is gained, the implanted markers can be totally eliminated, which can avoid an invasive procedure while reducing the cost associated with the procedure. The topic of whether the quality of KV‐CBCT is adequate for soft‐tissue alignment to eliminate the implant markers has been debated.^19^ Despite non‐ideal dosimetric coverage, our clinical outcome study for this group of patients reported a favorable rate of biochemical control and acceptably low rate of acute and long‐term GU and GI toxicities.^20^

## 5 CONCLUSIONS

Following alignment of fiducial markers with kV x ray, additional translational shifts were detected by kV‐CBCT and were clinically applied in 22 of 84 daily fractions, which were distributed in 50% of the observed patients. Our finding suggests that marker‐based alignment alone may not be sufficient, and CBCT based soft‐tissue alignment with implanted markers to guide soft‐tissue alignment can improve the tumor coverage for prostate SBRT. Future clinical outcome studies are needed to confirm our observations. Another ongoing study is to determine whether there is a correlation between the fullness of the bladder and magnitude of the prostate rotation.

## CONFLICTS OF INTEREST

The authors have no relevant conflicts of interest to disclose.
