# Supplementary material for: Volumetric‐based image guidance is superior to marker‐based alignments for stereotactic body radiotherapy of prostate cancer
Source: J Appl Clin Med Phys. 2018 Feb 15;19(2):198–203. doi: 10.1002/acm2.12280 (PMC5849820; doi:10.1002/acm2.12280)
Supplement: Supplementary file 1 [file ACM2-19-198-s001.docx]

**References**

^1^ Ishiyama H, Teh BS, Lo SS, et al. Stereotactic body radiation therapy for prostate cancer. Futur. Oncol 2011; **7**(9): 1077–1086.

^2^ Kim DWN, Straka C, Cho LC, Timmerman RD. Stereotactic Body Radiation Therapy for Prostate Cancer: Review of Experience of a Multicenter Phase I/II Dose-Escalation Study. Front. Oncol. 2014; **4**(November): 1–7.

^3^ Katz AJ, Santoro M, Diblasio F, Ashley R. Stereotactic body radiotherapy for localized prostate cancer: Disease control and quality of life at 6 years. Radiat. Oncol. 2013; **8**(1): 1–8.

^4^ Both S, Wang KKH, Plastaras JP, et al. Real-time study of prostate intrafraction motion during external beam radiotherapy with daily endorectal balloon. Int. J. Radiat. Oncol. Biol. Phys. 2011; **81**(5): 1302–1309.

^5^ Court LE, D’Amico A V., Kadam D, Cormack R. Motion and shape change when using an endorectal balloon during prostate radiation therapy. Radiother. Oncol. 2006; **81**(2): 184–189.

^6^ Jones BL, Gan G, Diot Q, Kavanagh B, Timmerman RD, Miften M. Dosimetric and deformation effects of image-guided interventions during stereotactic body radiation therapy of the prostate using an endorectal balloon. Med. Phys. 2012; **39**(6): 3080.

^7^ Smeenk RJ, Louwe RJW, Langen KM, et al. An endorectal balloon reduces intrafraction prostate motion during radiotherapy. Int. J. Radiat. Oncol. Biol. Phys. 2012; **83**(2): 661–669.

^8^ Wang KKH, Vapiwala N, Deville C, et al. A study to quantify the effectiveness of daily endorectal balloon for prostate intrafraction motion management. Int. J. Radiat. Oncol. Biol. Phys. 2012; **83**(3): 1055–1063.

^9^ Amro H, Hamstra DA, McShan DL, et al. The dosimetric impact of prostate rotations during electromagnetically guided external-beam radiation therapy. Int. J. Radiat. Oncol. Biol. Phys. 2013; **85**(1): 230–236.

^10^ Barney BM, Lee RJ, Handrahan D, Welsh KT, Cook JT, Sause WT. Image-guided radiotherapy (IGRT) for prostate cancer comparing kV imaging of fiducial markers with cone beam computed tomography (CBCT). Int. J. Radiat. Oncol. Biol. Phys. 2011; **80**(1): 301–305.

^11^ Deegan T, Owen R, Holt T, et al. Assessment of cone beam CT registration for prostate radiation therapy: Fiducial marker and soft tissue methods. J. Med. Imaging Radiat. Oncol. 2015; **59**(1): 91–98.

^12^ Moseley DJ, White EA, Wiltshire KL, et al. Comparison of localization performance with implanted fiducial markers and cone-beam computed tomography for on-line image-guided radiotherapy of the prostate. Int. J. Radiat. Oncol. Biol. Phys. 2007; **67**(3): 942–953.

^13^ Ahunbay EE, Peng C, Holmes S, Godley A, Lawton C, Li XA. Online adaptive replanning method for prostate radiotherapy. Int. J. Radiat. Oncol. Biol. Phys. 2010; **77**(5): 1561–1572.

^14^ Feng Y, Castro-Pareja C, Shekhar R, Yu C. Direct aperture deformation: An interfraction image guidance strategy. Med. Phys. 2006; **33**(12): 4490.

^15^ Wu C, Jeraj R, Olivera GH, Mackie TR. Re-optimization in adaptive radiotherapy. Phys. Med. Biol. 2002; **47**(17): 3181–3195.

^16^ Wu QJ, Thongphiew D, Wang Z, et al. On-line re-optimization of prostate IMRT plans for adaptive radiation therapy. Phys. Med. Biol. 2008; **53**(3): 673–91.

^17^ Owen R, Kron T, Foroudi F, Milner A, Cox J, Duchesne G. Interfraction prostate rotation determined from in-room computerized tomography images. Med. Dosim. 2011; **36**(2): 188–194.

^18^ Shang Q, Sheplan Olsen LJ, Stephans K, Tendulkar R, Xia P. Prostate rotation detected from implanted markers can affect dose coverage and cannot be simply dismissed. J. Appl. Clin. Med. Phys. 2013; **14**(3): 4262.

^19^ Njeh CF, Parker BC, Orton CG. Implanted fiducial markers are no longer needed for prostate cancer radiotherapy. Med. Phys. 2017;

^20^ Kotecha R, Djemil T, Tendulkar RD, et al. Dose-Escalated Stereotactic Body Radiation Therapy for Patients With Intermediate- and High-Risk Prostate Cancer: Initial Dosimetry Analysis and Patient Outcomes. Int. J. Radiat. Oncol. Biol. Phys. 2016; **95**(3): 960–964.
